# Exploring Uncertainty in Canine Cancer Data Sources Through Dasymetric Refinement

**DOI:** 10.3389/fvets.2019.00045

**Published:** 2019-02-26

**Authors:** Gianluca Boo, Stefan Leyk, Sara I. Fabrikant, Ramona Graf, Andreas Pospischil

**Affiliations:** ^1^Department of Geography, University of Zurich, Zurich, Switzerland; ^2^Collegium Helveticum, University of Zurich, ETH Zurich, Zurich, Switzerland; ^3^WorldPop, Department of Geography and Environment, University of Southampton, Southampton, United Kingdom; ^4^Department of Geography, University of Colorado, Boulder, CO, United States; ^5^Digital Society Initiative, University of Zurich, Zurich, Switzerland

**Keywords:** geographic correlation studies, canine cancer incidence, cancer underascertainment, spatial data aggregation, dasymetric refinement

## Abstract

In spite of the potentially groundbreaking environmental sentinel applications, studies of canine cancer data sources are often limited due to undercounting of cancer cases. This source of uncertainty might be further amplified through the process of spatial data aggregation, manifested as part of the modifiable areal unit problem (MAUP). In this study, we explore potential explanatory factors for canine cancer incidence retrieved from the Swiss Canine Cancer Registry (SCCR) in a regression modeling framework. In doing so, we also evaluate differences in statistical performance and associations resulting from a dasymetric refinement of municipal units to their portion of residential land. Our findings document severe underascertainment of cancer cases in the SCCR, which we linked to specific demographic characteristics and reduced use of veterinary care. These explanatory factors result in improved statistical performance when computed using dasymetrically refined units. This suggests that dasymetric mapping should be further tested in geographic correlation studies of canine cancer incidence and in future comparative studies involving human cancers.

## Introduction

Following the recent worldwide escalation of incidence, human cancer registration was initially introduced in hospitals at the beginning of the twentieth century and progressively implemented in several national surveillance programs (1–3). As a result, human cancer registries have become an essential data source for epidemiological research, confirming etiologies such as specific lifestyles, behavioral factors, and environmental exposures (3, 4). Despite these significant achievements, the understanding of relationships between human cancers and environmental risk factors remains limited for several reasons. One of these reasons is of specific relevance to this study—exposure measurement error (5, 6), which is known to be one of the primary sources of uncertainty in epidemiological studies (7).

One of the main causes of measurement error lies in the long latency period of many cancers and the difficulty to systematically assess exposure to environmental risk factors over time, especially for populations with dynamic mobility patterns (7, 8). To partly overcome this analytical limitation, ongoing research is engaging in comparative studies of cancers in companion animals (9, 10). Similar to the iconic canary in a coal mine, companion animals could be used as sentinels for environmental risk factors at the household level because they share the same living environment with their owners and have shorter latency periods than humans (11, 12). In some instances, health data of companion animals could potentially serve as accurate and timely indicators of exposure to environmental risk factors for human cancers (12). The relevance of these sentinel applications is supported by comparative research linking similar cancer types in dogs and humans to shared environmental risk factors (13–17).

These linkages have also been highlighted by spatial epidemiological studies that incorporate the geographic perspective into “the design and analysis of the distribution, determinants, and outcomes” (18) of canine cancers. These studies ranged from disease mapping (19) to cluster detection (20), and geographic correlation studies (21). For instance, through cluster analysis, similar geographic distributions of selected cancer types in dogs and humans have been identified in Michigan, USA (20). However, comparative studies in the domain of spatial epidemiology are limited in numbers, because few canine cancer registries are currently active, and those that exist, involve different inclusion criteria and incompatible collection methods. Such collection-related issues, together with unreliable information on dog demographics and undercounting of canine cancer cases, are persistent sources of uncertainty challenging spatial epidemiological research (22–24).

To advance the understanding of some of the uncertainties presented above, we examine the Swiss Canine Cancer Registry (SCCR)—a data source compiled for future comparative studies of canine and human cancers. These future studies are meant to inform cancer research as well as planning and evaluation of cancer prevention programs in Switzerland (25). Although the SCCR was designed to overcome collection- and classification-related issues, uncertain demographic characteristics of the at-risk dog population and potential undercounting of canine cancer cases persist (26–29). Undercounting comprises underreporting and underascertainment—two distinct but often confused phenomena (30). While underreporting takes place when the result of a performed diagnostic examination is not reported in the data source, underascertainment occurs when the diagnostic examination has not been performed at all (31, 32).

While underreporting is considered a marginal issue in the SCCR data (25, 33, 34), we explore potential underascertainment of canine cancer cases, in other words, undercounting related to the fact that the dog owner might not seek veterinary care for canine cancer diagnosis. Given this particular aim, methods to assess undercounting in canine cancer registry data, such as the capture-recapture method cannot be easily employed because information about the missing diagnostic examination cannot be retrieved from any other data source (30–32). For these reasons, our assessment is performed in a regression analysis framework to explore statistical associations between canine cancer incidence computed across Swiss municipalities and selected explanatory variables accounting for known demographic risk factors and potential underascertainment of canine cancer cases (26–29, 33, 34).

Because the SCCR data are enumerated within municipal units, our assessment is also likely to be affected by issues of spatial data aggregation, manifested as part of the modifiable areal unit problem (MAUP) (35, 36). Several studies demonstrated effects of spatial data aggregation related to the MAUP in different spatial epidemiological applications, such as disease mapping (37–40), cluster detection (41–44), and geographic correlation studies (8, 45–47). To assess uncertainty in the SCCR data and to explore effects of spatial data aggregation, we contrast regression models based on two enumeration types—municipal units and dasymetrically refined units, comprising only the portion of residential land within the municipal unit (29). These units are used to spatially aggregate canine cancer incidence and the explanatory factors included in the regression models.

This analytical framework is meant to explore uncertainty related to potential underascertainment of canine cancer cases and effects of spatial data aggregation on the statistical associations and goodness-of-fit. Given that canine cancer data sources have rarely been studied within their geographic context, we anticipate that the results of this study are an essential stepping stone to foster a geographic perspective into future comparative studies of canine and human cancers.

## Materials and Methods

### Materials

#### Swiss Canine Cancer Registry

The SCCR stores canine cancer cases from 1955 to 2008 for the entire country of Switzerland. The registry has been retrospectively assembled by the Collegium Helveticum Zurich for future comparative studies of canine and human cancers and is in the process of being updated to include the most recent years (33, 34). To date, the SCCR comprises 121,936 diagnostic records issued from post-mortem and biopsy samples, which have been collected by the Vetsuisse faculties of veterinary pathology of Berne and Zurich. It additionally includes the analysis of biopsy samples performed by a private diagnostic laboratory, located on the outskirts of Zurich (33, 34). The examination methods adopted by these diagnostic laboratories have been discussed extensively elsewhere (25). The diagnostic data have been systematically enumerated at the municipal level because residential addresses were not consistently reported due to different imputation strategies adopted by the diagnostic institutes in the past. Based on the residential postcode, more than 99.9% of the diagnostic data have been successfully allocated to a municipal unit, while the remaining 0.1% with wrong or missing postal codes have been discarded.

In this study, we use the 3,611 diagnostic records collected during the year 2008, as previous research has shown that data quality perceptibly decreases for earlier years because the available diagnostic methods were less accurate (33, 34). We did not exclude any cancer cases from the analysis. Following the ICD-O-3 classification, the diagnosed cancers are the malignant forms of odontogenic neoplasia, trophoblastic tumors, epithelial tumors, germ cell tumors, lymphangioma, lymphangiosarcoma, lymphoid tumors, melanoma, mesenchymal tumors, skeletal tumors, neural tumors, gonadal tumors as well as unspecified tumors (33, 34). We developed a retrospective study of the SCCR data because dogs age up to five times faster than humans (25). As a consequence of a compressed lifespan, canine cancers develop much faster than human cancers (25, 33). A thorough assessment of a canine cancer incidence in 2008 is thus expected to be relevant for future comparative studies of canine and human cancers.

#### Demographic Risk Factors

We access the Swiss dog population census, as compiled by the Animal Identity Service AG (48), to produce demographic explanatory variables based on the at-risk dog population in Switzerland for the year 2008. This census was enacted following the obligation of dog microchipping and registration established in 2006 (49). Presently, this is the most accurate source of information about the dog population living in Switzerland, as a recent expert evaluation confirmed 95% completeness for the year 2008, and this percentage is steadily increasing. According to this census, 496,689 dogs are recorded during the study period, resulting in a ratio of 6.54 dogs per 100 inhabitants (49).

Based on the Swiss dog population census, we compute variables describing the number of at-risk dogs, dog average age (in years), female dog ratio (in percent), and mixed breed ratio (in percent) at the municipal level. Mixed breed dogs are defined according to the standards of the Fédération Cynologique Internationale (FCI) used in the Swiss dog population census (49). While age and sex have similar associations to cancers in dogs and humans (50, 51), the different levels of cancer incidence among breeds (52, 53) could be a potential source of uncertainty in future comparative studies of canine and human cancers. For this purpose, computing demographic indicators as continuous variables (e.g., average age) instead of categorical variables (e.g., age classes) enable a more straightforward identification of potential mismatches with prior findings on demographic determinants of canine cancers, which are typically conducted in a non-geographic context.

#### Underascertainment of Canine Cancer Cases

Following previous studies of the SCCR data, we selected explanatory variables of urban character and socioeconomic status to account for different personal motivations or abilities to make use of veterinary care, and, thus, for potential underascertainment of canine cancer cases (26–29, 33, 34). Firstly, we estimate the urban character of municipalities, because the use of veterinary care is expected to be more frequent among dog owners in urban areas (26–29). This variable is computed based on human population density (in 1,000 people per square kilometer), using population census data at the municipal level for the year 2008. The census data can be accessed through the website of the Swiss Federal Statistical Office (54). Secondly, we assume that in municipalities characterized by higher socioeconomic status, dog owners are more likely to own financial means for regular veterinary check-ups potentially resulting in more frequent cancer diagnoses (26–29). As a consequence, we consider a surrogate to estimate the socioeconomic status of municipalities through national income tax information for 2008 (in 1,000 Swiss Francs per capita), which can be accessed through the Swiss Federal Tax Administration website (55).

Lastly, we derive a measure of distance to veterinary care (in kilometers), as we expect that greater road distance to veterinary practices would result in increased underascertainment of canine cancer cases (26–29). This variable is based on the addresses of the 938 veterinary practices active in 2013, which have been retrieved from the Swiss Yellow Pages website (56). We create an hectometric distance-grid (i.e., with a 100 × 100 meter spatial resolution) representing travel distances along roads to the nearest veterinary practice (57), using the Swiss road network in 2008, which has been extracted from the VECTOR25 data model of the Swiss Federal Office of Topography (58). Municipal-level travel distances to the nearest veterinary practice are then computed by averaging the distance-grid values intersecting each enumeration unit. We use the addresses of registered veterinary practices in 2013 because data for 2008 are not available, retrospectively. However, information issued by the Swiss Registry of Medical Professions confirms that changes in the number of licensed veterinarians within this period are negligible (59).

#### Enumeration Units

We spatially aggregate the SCCR data and the explanatory variables at the municipal level because this is the finest spatial resolution for which the data are available. For this purpose, we use the 2,350 Swiss municipal boundaries for the year 2014, as derived from the swissBOUNDARIES3D vector data model of the Swiss Federal Office of Topography (58). We retrieve municipal unit boundaries for 2014 because the SCCR data for all prior years have been systematically geocoded based on the units existing in 2014. To evaluate changes in model performance related to possible effects of spatial data aggregation, we carry out a dasymetric refinement of the enumeration units, based on the areal extent of residential land within each municipality (60).

The use of residential land as an ancillary variable for dasymetric refinement is connected with the assumption that dogs and humans share the same living environment (29). We derive residential land data from the building and dwelling survey conducted by the Swiss Federal Statistical Office in 2014. The data is available as an hectometric grid (i.e., with a 100 × 100 meter spatial resolution), where grid cells are classified as residential land if they intersect the centroid of at least one residential building. The survey retrieves information on characteristics and geographic coordinates of the buildings from the Federal Register of Buildings and Dwellings (RBD) (54). We use more recent information on residential land because data for 2008 is not currently available. However, differences between the corresponding years are reported to be minimal, because of the increasing densification of residential land parcels, especially in peri-urban areas (54). For this reason, using the building and dwelling survey data for 2014 is seen as an acceptable compromise.

### Methods

#### Dasymetric Refinement of Enumeration Units

We employ a dasymetric-mapping framework to evaluate improvements in our models of canine cancer incidence that could be linked to reduced effects of spatial data aggregation. These effects, often described under the term of MAUP, influence statistical analyses using enumerated data because summary statistics may change according to the shape and areal extent of the enumeration unit (35, 36). Spatial data aggregation further implies that the aggregated data are homogeneously distributed within the enumeration units and that sharp changes of summary statistics occur across their boundaries characteristics—two of the underlying assumptions of the choropleth mapping method (60–62). However, choropleth maps can produce unrealistic spatial distributions of aggregated data in a spatial epidemiology context because populations, and thus diseases such as cancer, are usually not homogeneously distributed across the enumeration units (e.g., administrative units) (60).

**Figure 1:**
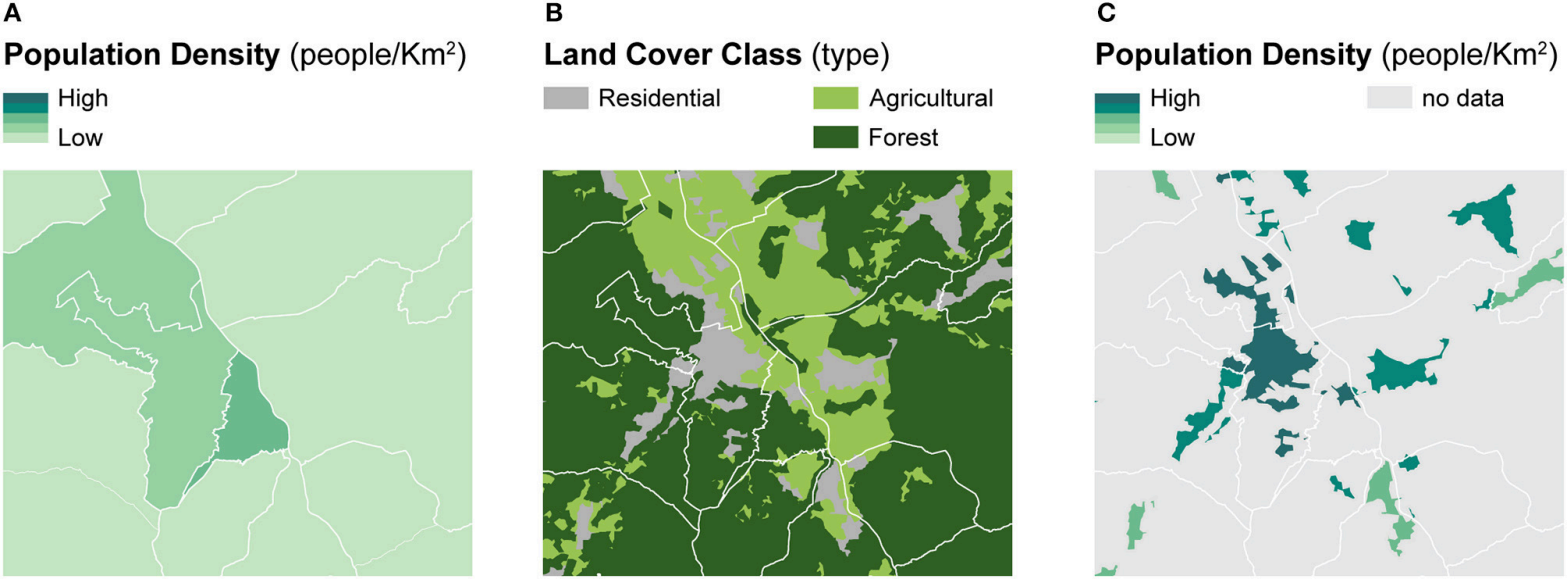
A framework for dasymetric refinement of population data within residential land. Example of binary dasymetric refinement of population data within residential land — **(A)** population density computed within administrative units is refined based on **(B)** the location of residential land to recompute **(C)** population density within dasymetrically refined units.

Dasymetric mapping is a cartographic method designed to produce more accurate spatial distributions of enumerated data with respect to geographic context using ancillary spatial variables. The ancillary variables, typically linked to population characteristics, are assumed to be related to the geographic distribution of the phenomenon of interest—which is often linked to population—more accurately (60–62). To highlight the differences between dasymetric mapping and choropleth mapping, Figure 1 illustrates the dasymetric refinement of population data within administrative units using the portion of residential land as a binary ancillary variable (Figure 1B) (60). Compared to the choropleth map based on administrative units (Figure 1A), the dasymetric map (Figure 1C) produces more accurate spatial distributions of the population densities within the enumeration units (60). Importantly, this sort of dasymetric refinement is constrained by the pycnophylactic property, implying that population counts of dasymetrically refined units should maintain the same total values as the original enumeration units (63).

Various kinds of data have been tested as ancillary variables for dasymetric refinement. For example, land cover (62, 64, 65), road density (66), remote sensing imagery (67, 68), parcel data (69–71), address points (67) and dwelling survey data (29) have been employed to refine the geographic distribution of populations within the original enumeration units.

The hectometric cells representing residential land are allocated to municipal units according to the location of their cell centroid. Once allocated to a municipality, the residential hectare cells are dissolved, and the resulting areal extents are employed as refined enumeration units. These dasymetrically refined units are finally used to enumerate canine cancer incidence and explanatory variables implemented in the regression models. However, the only two differences between regression models based on municipal units and those based on dasymetrically refined units can be found in the explanatory variables involving recomputed density and distance explanatory variables. This is because only these variables change according to the modified areal extent and location of the enumeration units. While the refinement of density variables is a natural application of dasymetric mapping (60–62), the recomputation of distance variables involves a change of support (i.e., downscaling from municipal units to their portion of residential land), which is not subject to the pycnophylactic property (72, 73). In both cases, the impact of dasymetric refinement on model associations and goodness-of-fit will be central and inform about potential improvements that could be related to reduced effects of spatial data aggregation.

#### Regression Models of Canine Cancer Incidence

Geographic correlation studies assess the relationships between disease incidence or mortality and the occurrence of potential demographic and environmental risk factors within their geographic context (18). Given that we are assessing count data for a relatively rare outcome, we use a Poisson regression as a baseline model to fit observed canine cancer incidence (y) across Swiss municipalities in 2008 (74). Dog Population is used as an offset (*e*), a constant of proportionality to account for the underlying at-risk dog population and compute incidence rates. Canine cancer incidence rates are fit through the following explanatory variables (*x*)—Dog Average Age (in years), Female Dog Ratio (in percent), Mixed Breed Ratio (in percent), Average Income Tax (in 1,000 Swiss Francs per capita), Human Population Density (in 1,000 people per square kilometer), and Distance to Veterinary Care (in kilometers). These explanatory variables are not centered to facilitate the interpretation of the resulting multiplicative coefficients (β) using the original reference units, but they are systematically tested for potentially induced multicollinearity (75, 76). For this purpose, we use the square root value of the variance inflation factor (SQVIF) because values >2.0 are reported to be critical to coefficient estimation (76). The equation below shows the formula of our baseline Poisson model, with regression to the mean of the data (μ_i_), where α is the intercept and β the multiplicative coefficients estimated for each explanatory variable reported above (77).

$$\begin{array}{l} y_{i}\sim \;Poisson\;\left(\mu_{i}\right)\\ log\left(\mu_{i}\right)=\alpha +\overset{K}{\underset{1=k}{\sum }}\beta_{K}x_{i,K}+log\left(e\right) \end{array}$$

This regression analysis framework is meant to explore uncertainty in the SCCR data and its impact on statistical associations, interpreted as multiplicative effects [i.e., log(β)] for predicting canine cancer incidence (*y*). Significant (α = 0.05) associations between canine cancer incidence and the explanatory variables accounting for potential underascertainment of canine cancer cases (i.e., Average Income Tax, Human Population Density, and Distance to Veterinary Care) will thus be central to the assessment of uncertainty in the SCCR data (26–29). Furthermore, the associations between canine cancer incidence and dog demographic risk factors (i.e., Dog Average Age, Female Dog Ratio, and Mixed Breed Ratio) can also highlight uncertainty when compared with prior findings on demographic determinants associated with canine cancers (52, 53, 78, 79). Such an analytical framework also allows for the assessment of changes in model associations and goodness-of-fit between the two enumeration types as Human Population Density and Distance to Veterinary Care are recomputed after the dasymetric refinement.

Considering that the Poisson model is based on the highly restrictive assumption of equidispersion (i.e., the variance is equal to the mean of the incidence data) (80), we compare four different regression models for count data—(A) Poisson model, (B) Poisson model with zero-inflation extension, (C) negative binomial model, and (D) negative binomial model with zero-inflation extension (80, 81). While the negative binomial models (C, D) relax the assumption of equidispersion by accounting for a variance greater than the mean (i.e., overdispersion), the zero-inflation extensions (B, D) model potential excess zeros in a separate logistic regression model with binary outcome (i.e., zero vs. non-zero counts) (80, 81). Model equations and directed acyclic graphs (DAGs) for the different models can be found in the Supplementary Material. Modeling these excess zeros separately has the advantage of providing insights into false zeros, associated with the structure of the SCCR data. To avoid model overspecification, we first implement all the explanatory variables presented above in the zero-inflation extensions, but we finally retain only the significant (α = 0.05) ones.

We first assess the goodness-of-fit of the regression models through the Akaike information criterion (AIC) (82). The lowest AIC measure indicates the highest goodness-of-fit and allows comparison of the goodness-of-fit of each of the regression models based on municipal units and dasymetrically refined units because the sample size remains the same. This comparison involves systematic pairwise relative-likelihood assessments of the probability that a model minimizes the estimated information loss similarly to the model with the lower AIC (83). To assess the significance (α = 0.05) of improvement of one model over another, we also perform systematic pairwise likelihood-ratio tests (84). This form of comparison is meant to overcome the use of the Vuong test (83, 84), as several concerns about its validity have been recently raised (85, 86). In addition to these assessments of goodness-of-fit, we examine changes in the associations between the models based on the two enumeration types. This assessment contrasts significant (α = 0.05) multiplicative effects resulting from the coefficient estimates and their effect sizes, estimated through the percentage of deviance reduction (80, 81).

Data processing, analysis, visualization, and statistical modeling were carried out using RStudio Desktop 1.1.463 (87). The following R packages were used in this study—gdistance (88), ggplot2 (89), maptools (90), plyr (91), pscl (92), reshape (93), rgdal (94), and sandwich (95).

## Results

### Contrasting Municipal and Dasymetrically Refined Units

**Figure 2:**
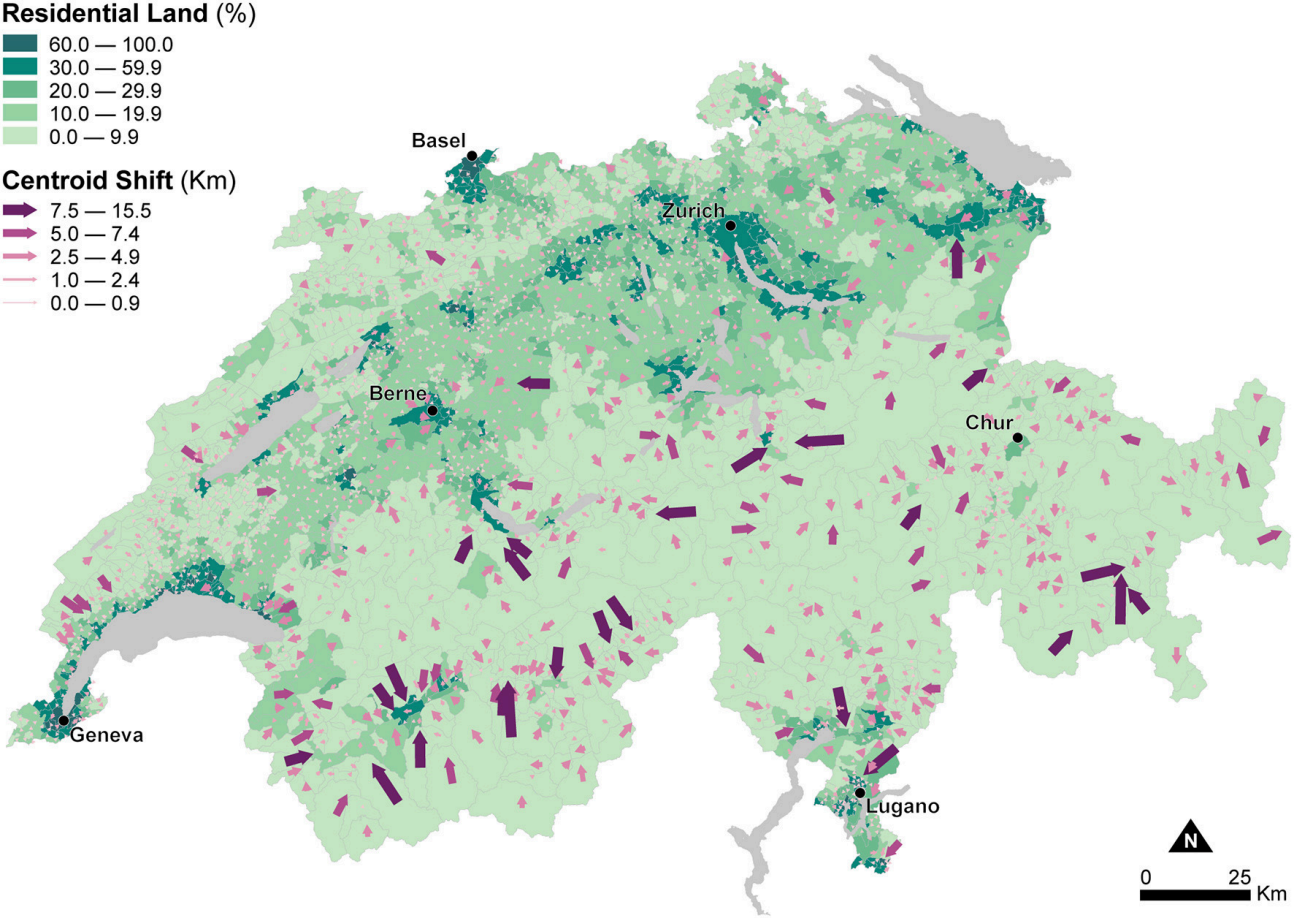
Effects of dasymetric refinement of the municipal units — changes of spatial extent (part of residential land) and centroid displacements (shift to the centroid of the residential land). The data is classified according to the fixed classes classification method.

Figure 2 shows the portion of residential land within municipal units to inform about changes in the areal extent of enumeration units due to dasymetric refinement. These changes in areal extent impact the recomputation of density indicators—in this study Human Population Density—used as explanatory variables in the models of canine cancer incidence. Figure 2 indicates that substantial differences in the areal extent occur in the Alps and the Jura Mountains, which show very low residential land proportions, mostly <10.0%. In contrast, higher residential land proportions, between 10.0 and 59.9%, generally characterize the Central Plateau, with peak proportions exceeding 60.0% for the larger urban agglomerations like Zurich, Geneva, or Basel.

Figure 2 also shows changes in the relative location of the enumeration unit centroids due to the change of support resulting from dasymetric refinement. The width and direction of the purple arrows symbolize the magnitude and direction of displacement of the centroids from the municipal units (base of the arrow) to the dasymetrically refined units (point of the arrow). These changes in relative location impact the recomputation of distance indicators—in this study travel Distance to Veterinary Care along roads—used as explanatory variables in the models of canine cancer incidence. Again, the largest centroid shifts, between 2.5 and 15.5 km, occur in the sparsely populated Alps, while centroid shifts are much smaller, between 0.0 and 2.4 km, in the densely populated Central Plateau. As mentioned, the changes in the areal extent and relative location of the enumeration units highlighted in Figure 2 influence the recomputation of Human Population Density and Distance to Veterinary Care, and how their use as explanatory variables for modeling canine cancer incidence is likely to modify the estimated associations and goodness-of-fit.

**Figure 3:**
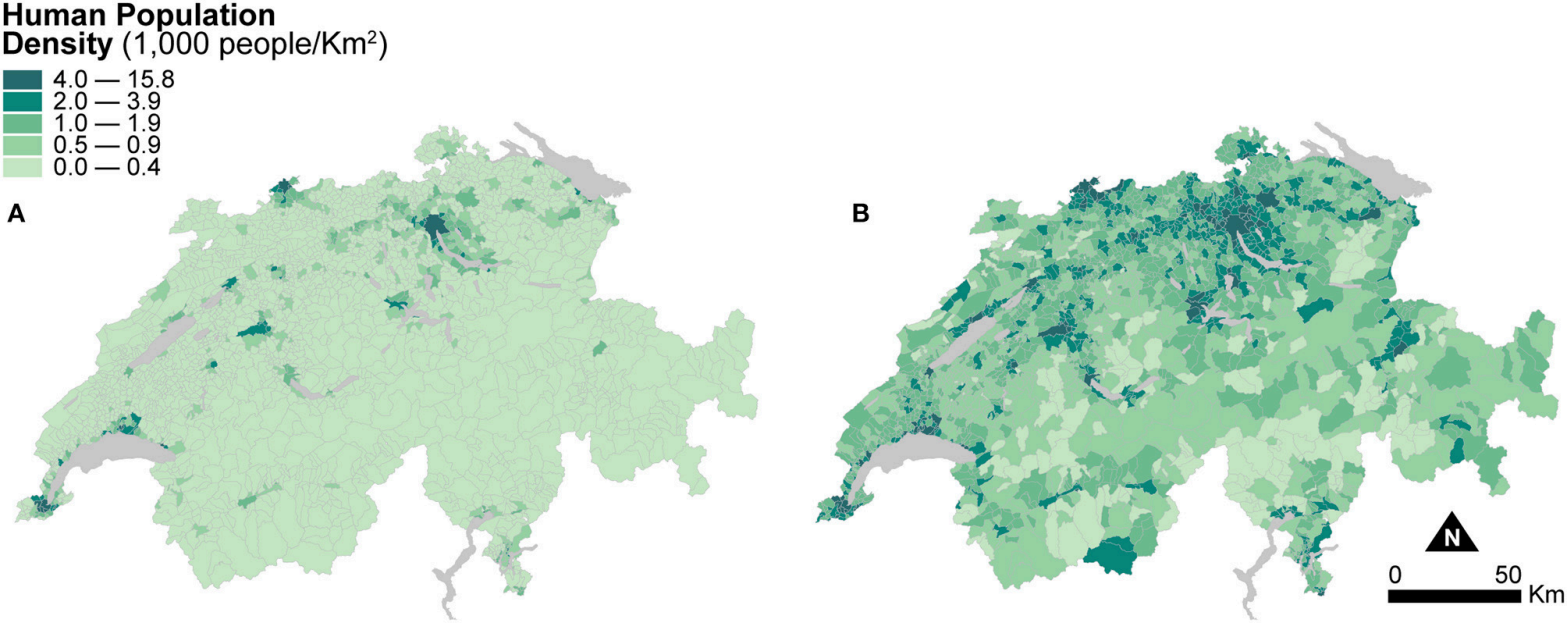
Human population density indicators resulting from the two enumeration types — **(A)** municipal units and **(B)** dasymetrically refined units. For better visual comparison, the indicator recomputed after dasymetric refinement is also presented in a choropleth fashion. The data is classified according to the quantile classification method applied to the dasymetrically refined units.

Figure 3 shows two maps of human population density at the municipal level. The first one is computed based on the areal extent of municipal units (Figure 3A), and the second one is based on the areal extent of dasymetrically refined units (Figure 3B). For better visual comparison, in Figure 3B, the recomputed population densities after dasymetric refinement are also shown in a choropleth fashion. The use of dasymetrically refined units yields substantially higher human population densities because the areal extent for density recomputation is reduced to the portion of residential land within municipal units. This effect is notable in the mountainous regions and in most municipalities of the flat Central Plateau, where human population densities also perceptibly increase. Human Population Density recomputed using dasymetrically refined units is thus likely to produce more accurate spatial distributions and more robust associations in the models of canine cancer incidence.

**Figure 4:**
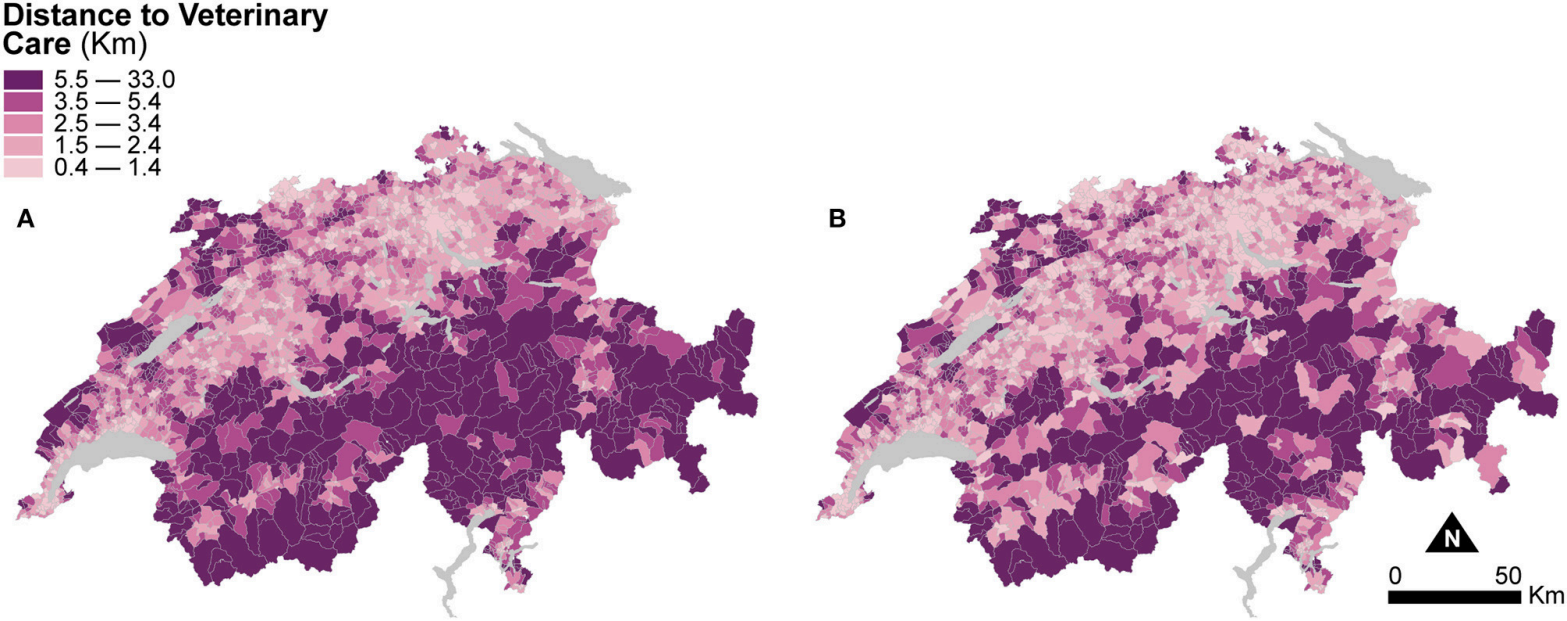
Distance to veterinary care indicators resulting from the two enumeration types — **(A)** municipal units and **(B)** dasymetrically refined units. For better visual comparison, the indicator recomputed after dasymetric refinement is also presented in a choropleth fashion. The data is classified according to the quantile classification method applied to the dasymetrically refined units.

Figure 4 shows two maps of road distances to the closest veterinary practice averaged at the municipal level. The first is computed based on the areal extent of the municipal units (Figure 4A), and the second is based on the areal extent of dasymetrically refined units (Figure 4B). Similar to Figure 3, for direct visual comparison, Figure 4 shows both maps in choropleth fashion. Despite the change of support, averaged distances to veterinary care are similar in both maps. The mean distance to veterinary care is 4.05 km (*SD* = 3.56) for municipal units, slightly higher than the mean distance of 3.63 km (*SD* = 3.43) for dasymetrically refined units. In both cases, the important spread suggests a persisting impact of large distances to veterinary care, as shown in the Alps and the Jura Mountains.

### Modeling Canine Cancer Incidence

**Figure 5:**
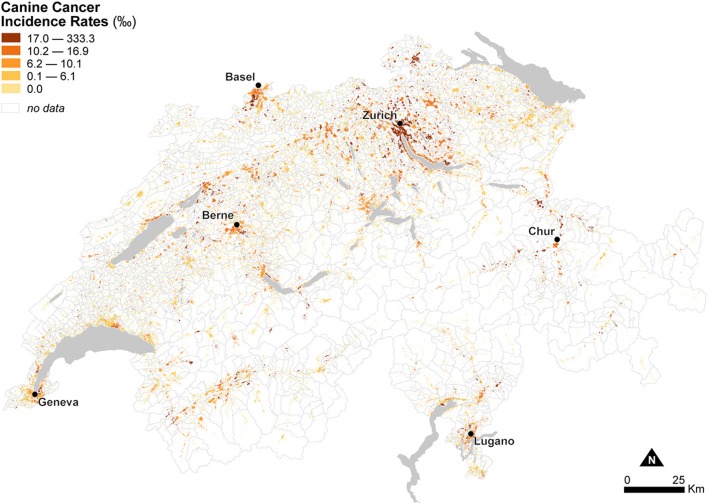
Observed canine cancer incidence rates across Swiss municipalities in 2008. The data is classified according to the quantile classification method and mapped using dasymetrically refined units.

Figure 5 shows the spatial distribution of observed canine cancer incidence rates at the municipal level in Switzerland for the year 2008 using dasymetrically refined units. Canine cancer incidence rates seem to exhibit a particular geographic configuration, with high rates in the German-speaking northeast of the Central Plateau compared to low to mixed rates in the French-speaking, western part of the country. In the Alps and the Jura Mountains, rates are mostly very low or even zero. Figure 5 also provides visual support for our dasymetric framework, as it enables a more meaningful interpretation of the spatial distribution of canine cancer incidence rates.

To provide an in-depth insight into uncertainty in the SCCR data, we fit our four regression models for count data using a (A) Poisson model, (B) Poisson model with zero-inflation extension, (C) negative binomial model, and (D) negative binomial model with zero-inflation extension—for the two types of enumeration units (i.e., municipal units and dasymetrically refined units). In these models, the square root of the variance inflation factor (SQRVIF) values is consistently below 2.0. The zero-inflated extensions in models (B) and (D) implement only Dog Average Age because this is the only significant (*P* < 0.05) explanatory variable in the logistic model component. Table 1 presents the AIC measures for the four regression models and both types of enumeration units.

**Table 1:** AIC measures for the different regression models based on the two enumeration types — **(A)** municipal units and **(B)** dasymetrically refined units.

**Regression model**	**(A) Municipal units**	**(B) Dasymetrically refined units**
Poisson	6449.3	6419.7
Negative binomial	5930.2	5910.7
Poisson with zero inflation	6243.2	6223.5
Negative binomial with zero inflation	5894.5	5878.2

Table 2 presents the results of the pairwise likelihood-ratio tests to determine model improvements. In the likelihood ratio test, a positive χ^2^ value rejects Model 1, and the significance level of the test is reported in parentheses. The relative likelihood assessments consistently endorse the results of the likelihood-ratio tests with values of 0.00. In each row, the model with the best goodness-of-fit, both according to the relative likelihood and the likelihood-ratio, is highlighted in bold. These tests suggest that the negative binomial model with zero-inflation extension (D) outperforms the other models for both types of enumeration units. On the one hand, this is because the model shows the lowest AIC measure and the pairwise relative likelihood assessments confirm that the likelihood that the other models can compete in minimizing the information loss is extremely low (i.e., 0.00). On the other hand, the likelihood-ratio test shows that this improvement is significant (*P* < 0.05).

**Table 2:** Pairwise likelihood-ratio tests comparing the different regression models based on the two enumeration types — **(A)** municipal units and **(B)** dasymetrically refined units. The *P* value of the tests is consistently <0.05. The best model is highlighted in bold.

**Model 1**	**Model 2**	**(A) Municipal units**	**(B) Dasymetrically refined units**
Poisson	**Negative binomial**	521.1	511.0
Poisson	**Poisson with zero inflation**	210.1	200.2
Negative binomial	**Poisson with zero inflation**	311.0	310.8
Negative binomial	**Negative binomial with zero inflation**	39.7	36.5
Poisson with zero inflation	**Negative binomial with zero inflation**	350.7	347.3

Table 3 shows the coefficient estimates and the percentage of deviance reduction related to each explanatory variable for the negative binomial model with zero-inflation extension (4) using both types of enumeration units. The coefficient estimates suggest that Dog Average Age exhibits negative associations, such that for each increasing year of age, the incidence rates decreased by 17.3% (for municipal units) and 18.9% (for dasymetrically refined units). Conversely, Female Dog Ratio and Mixed Breed Ratio both produce positive associations, as for each increasing percentage unit of female dogs and mixed-breed dogs, the incidence rates increase by 1.0% (for municipal units) and 2.0% (for dasymetrically refined units) and 3.1% (for municipal units) and 2.0% (for dasymetrically refined units), respectively.

**Table 3:** Coefficients for the negative binomial model with zero-inflation extension based on the two enumeration types — **(A)** municipal units and **(B)** dasymetrically refined units.

	**(A) Municipal units**				**(B) Dasymetrically refined units**			
	**Estimate**	**Standard error**	***P***	**Deviance reduction (%)**	**Estimate**	**Standard error**	***P***	**Deviance reduction (%)**
**NEGATIVE BINOMIAL**								
Dog Average Age	−0.19	0.04	< 0.05	20.40	−0.21	0.05	< 0.05	13.86
Female Dog Ratio	0.01	0.01	< 0.05	3.76	0.02	0.01	< 0.05	5.18
Mixed Breed Ratio	0.03	0.00	< 0.05	20.40	0.02	0.01	< 0.05	18.39
Average Income Tax	0.11	0.02	< 0.05	21.53	0.11	0.02	< 0.05	23.06
Human Population Density	0.04	0.03	0.23	0.75	0.08	0.01	< 0.05	9.33
Distance to Veterinary Care	−0.04	0.11	< 0.05	8.76	−0.03	0.11	< 0.05	4.92
**ZERO INFLATION**								
Dog Average Age	−3.61	0.61	< 0.05	24.41	−3.69	0.63	< 0.05	25.26

Average Income Tax and Human Population Density both exhibit positive associations, showing that for each 1,000 CHF per capita and 1,000 individuals per square kilometer, the incidence rates increase by 11.6% (both for municipal units and dasymetrically refined units) and 4.1% (for municipal units) and 8.3% (for dasymetrically refined units), respectively. However, the latter association is not significant in the model based on municipal units (*P* = 0.23). Distance to Veterinary Care exhibits negative associations, indicating that for each additional kilometer, the incidence rates decrease by 3.0% (for municipal units) and 3.9% (for dasymetrically refined units). Lastly, in the zero-inflation extension of the models, Average Age showed negative associations, suggesting that for each increasing year, the odds of observing zero incidence rates decrease by 97.3% (for municipal units) and 97.5% (for dasymetrically refined units).

Table 3 also provides insights into how goodness-of-fit is related to effects of spatial data aggregation. As mentioned, the coefficient estimate for Human Population Density is significant (*P* < 0.05) only in the regression model using dasymetrically refined units. This explanatory variable also shows a higher percentage of deviance reduction when using dasymetrically refined units. In contrast, the coefficient estimate for Distance to Veterinary Care is significant (*P* < 0.05) in the regression models based on both types of enumeration units, and the percentage of deviance reduction even increases when using municipal units. Table 3 also shows that the recomputation of Human Population Density and Distance to Veterinary Care also influences other explanatory variables and the overall goodness-of-fit of the models. In particular, we can observe higher percentages of deviance reduction for the explanatory variables potentially related to underascertainment of canine cancer cases when using dasymetrically refined units.

Finally, we compare the overall goodness-of-fit of the regression models based on the two enumeration types through the relative likelihood and the likelihood-ratio test. The former shows that the regression model based on municipal units is very unlikely (i.e., 0.00) to compete in minimizing the information loss with the one based on dasymetrically refined units. The latter shows that the regression model based on dasymetrically refined units results in a significant improvement over the model based on municipal units (*P* < 0.05; χ^2^ = 16.3).

## Discussion

### Assessing Uncertainty in the Swiss Canine Cancer Registry

This study aimed at identifying and understanding sources of uncertainty in the SCCR data and their impact on models of canine cancer incidence. For this purpose, we examined two types of explanatory variables—those relating to dog demographic risk factors and those accounting for potential underascertainment of canine cancer cases. This analytical framework helped in reflecting on the uncertainty surrounding both the SCCR data and the statistical associations estimated in models of canine cancer incidence, in general.

Our results show that, in the negative binomial component of the models, most of the relationships between canine cancer incidence and demographic risk factors contrast with prior findings. These relationships are particularly critical, for instance, when negative associations between canine cancer incidence and Dog Average Age are implied. This unexpected finding is likely to reflect different personal motivations and abilities to make use of veterinary care, resulting in selective underascertainment of cancer cases in older canine populations (96). However, in contrast, such a phenomenon is not captured in the zero-inflation extension of the models, as Dog Average Age confirms prior findings on increasing canine cancer incidence in older dogs (78, 79). Uncertainty in the SCCR data is also suggested by the positive association between canine cancer incidence and Mixed Breed Ratio, which contradicts prior findings of higher canine cancer incidence among pure-breed dogs. Still, these results are difficult to compare because mixed breed dogs can have very different lifespans (52).

The relationships between canine cancer incidence and the variables accounting for potential underascertainment of canine cancer cases helped in reflecting personal motivations or abilities to make use of veterinary care, thus, explaining some of the contradicting statistical associations. Positive associations between cancer incidence and both Average Income Tax and Human Population Density confirmed prior findings suggesting that higher socioeconomic status and urban lifestyle involve more frequent uses of veterinary care, thus, a more consistent ascertainment of canine cancer cases (26–29). We also found a negative association between canine cancer incidence and Distance to Veterinary Care, confirming our expectation that greater road distances to veterinary care affect the motivations or abilities to make use of veterinary care, thus, an increased underascertainment of canine cancer cases (26–29). Furthermore, the effect sizes of the variables accounting for potential underascertainment of canine cancer cases and the goodness-of-fit of the model associated with the zero-inflation extension confirmed that underascertainment of canine cancer cases impacts the completeness of the SCCR data.

Compared with existing studies of the SCCR data (33, 34) and other canine cancer registries (50, 51) that have been carried out in a non-geographic context, the effect of underascertainment of canine cancer cases becomes prominent in this study because canine cancer incidence and explanatory variables are enumerated within Swiss municipal units. Using these enumeration units implies very high levels of underascertainment in places located within mountainous regions, such as the Alps and the Jura mountain range, potentially resulting in highly inaccurate canine cancer incidence estimates. For this reason, the sources of uncertainty highlighted through our analytical effort need careful consideration when developing future comparative studies of canine and human cancers based on the SCCR data and similar data sources. These future studies should focus on selected cancer types to include more targeted environmental risk factors, such as sun exposure for melanoma or environmental pollution for lung cancer. They should also test more complex spatial modeling techniques to include random effects, for instance, conditional autoregressive models (CAR) and to include measurement error components to adjust the expected incidence by the probability that a dog with cancer would be taken to a veterinarian (97, 98).

### Evaluating the Effects of Spatial Data Aggregation

To explore effects of spatial data aggregation on the models of canine cancer incidence, we refined the municipal units in a binary fashion using the areal extent of residential land (60–62). This was meant to better account for the specific geographic configuration of populated land within enumeration units because dasymetric refinement has been reported particularly effective in sparsely populated regions (61). Because of the changes in the areal extent and location of the enumeration units, the recomputation of density and distance explanatory variables was, thus, expected to impact statistical association in the models of canine cancer incidence (29).

Human Population Density produces a significant coefficient estimate (*P* < 0.05) only in the regression model using dasymetrically refined units, and this type of enumeration unit also exhibits a higher percentage of deviance reduction. This result confirms prior findings showing that dasymetric refinement can result in more accurate density variables (60–62) and, thus, more robust statistical associations with canine cancer incidence. In contrast, the multiplicative effects for Distance to Veterinary Care do not show any relevant change associated with dasymetric refinement, and the percentage of deviance reduction even decreases when using dasymetrically refined units. This finding suggests that if dasymetric refinement does not result in changes in average distance measures as in this study, statistical associations in models of canine cancer incidence will remain largely unaffected. Such a result highlights that change of support problems are not trivial and need in-depth consideration in geographic correlation studies (72, 73).

Nevertheless, when contrasting the goodness-of-fit of the models of canine cancer incidence based on the two enumeration types, we detected a systematic improvement associated with the use of dasymetrically refined units. This improvement suggests that dasymetric refinement could mitigate effects of spatial data aggregation when recomputing explanatory variables to be implemented in models of canine cancer incidence. For this reason, we contend that our analytical framework provides relevant insights both for future comparative studies of specific cancer types that involve spatially explicit environmental variables. For this reason, in future studies, we aim to further refine our dasymetric framework, for example, by testing additional ancillary variables and fishnets of different spatial resolutions (64, 65). This will provide more detailed analyses of scale effects across different spatial units and, in turn, support more effective strategies to cope with effects of spatial data aggregation.

### Fostering a Geographic Perspective Into New Cancer Research Practices

Spatial epidemiology traditionally focuses on the study of the distribution, determinant, and outcomes, among others, of cancer in humans (19). Within this discipline, existing studies of human cancer registries have shed light on risk factors associated with specific lifestyles, behavioral factors, and environmental exposures (3, 4). However, known analytical limitations related, for instance, to exposure measurement error require complementing the studies of human cancers with new research practices and data sources (9, 10). For this reason, in this study, we examined the SCCR—a unique data source that has been assembled for future comparative studies of canine and human cancers (25). Such a comparative approach aims, for example, to reduce measurement error by providing timely indications of exposure to environmental risk factors for human cancers (11, 12).

Despite the apparent benefits suggested by disease mapping (19), cluster detection (20), and geographic correlation studies (21), comparative research of canine and human cancers is currently challenged by uncertainties in the existing canine cancer registry data sources (22–24). Given the geographic dimension of these spatial epidemiological investigations, unexplored sources of uncertainty, connected with effects of spatial data aggregation were also expected to affect the estimation of statistical associations between canine cancer incidence and geographically explicit environmental risk factors (72, 73). For this reason, we complemented our investigation of underascertainment of canine cancer cases with an assessment of effects of spatial data aggregation in models of canine cancer incidence.

By contrasting statistical performance and associations estimated based on municipal and dasymetrically refined units, we emphasize the importance of the geographic context in the study of canine cancer incidence in Switzerland. Similar to other study areas, this country presents sharp variations in the distribution of human and canine populations, which are related to geographic context (e.g., mountainous vs. flat regions)—a setting in which dasymetric refinement can be highly beneficial (60–62). As a consequence, this technique produced a more accurate reflection of the distribution of canine cancer incidence and geographically explicit explanatory variables (i.e., human population density and distance to veterinary care). This results in a more reliable assessment of uncertainties in the SCCR data through the models of canine cancer incidence.

These findings enable us to contend that our study of canine cancer incidence advances the understanding of effects of spatial data aggregation in geographic correlation studies. Also, following the definition of spatial epidemiology, our findings further advocate for the systematic implementation of a geographic perspective into cancer research practices involving new and unexplored data sources.

## Ethics Statement

This study was carried out in accordance with the recommendations of the Swiss Animal Welfare Act (Tierschutzgesetz–TSchG) of 2005 and the Animal Welfare Ordinance (Tierschutzverordnung–TSchV) of 2008 as it does not involve the primary collection of experimental data.

## Author Contributions

RG collected and pre-processed the SCCR data. GB processed the data, developed and implemented the study design, interpreted the results, and wrote the first draft of the manuscript. SL edited the manuscript, contributed to the design, implementation, and interpretation of the results. SF and AP edited the manuscript and contributed to the interpretation of the results.

### Conflict of Interest Statement

The authors declare that the research was conducted in the absence of any commercial or financial relationships that could be construed as a potential conflict of interest.
