# Supplementary material for: Exploring Uncertainty in Canine Cancer Data Sources Through Dasymetric Refinement
Source: Front Vet Sci. 2019 Feb 26;6:45. doi: 10.3389/fvets.2019.00045 (PMC6399139; doi:10.3389/fvets.2019.00045)
Supplement: Supplementary file 1 [file Data_Sheet_1.PDF]

Supplementary Material

Below are presented the model equations and the directed acyclic graphs (DAGs) derived from Zeilis *et al.* (2008).

1. Poisson regression

Equation 1

$$\boldsymbol{y_i \sim Poisson\ (\mu_i)}$$

$$\boldsymbol{log(\mu_i) = \alpha + \sum_{1=k}^{K} \beta_K\, x_{i,K} + log(e)}$$

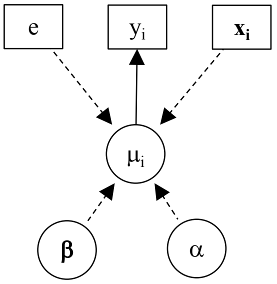

Figure 1. DAG for the Poisson regression model. Dashed lines represent deterministic relationships and solid line represent stochastic relationships. Rectangles represent data and circles model parameters. Vector of values are represented in bold.

2. Negative binomial regression

Equation 2

$$y_i \sim Negative\ Binomial\ (\mu_i, \theta)$$

$$log(\mu_i) = \alpha + \sum_{1=k}^{K} \beta_K\, x_{i,K} + log(e)$$

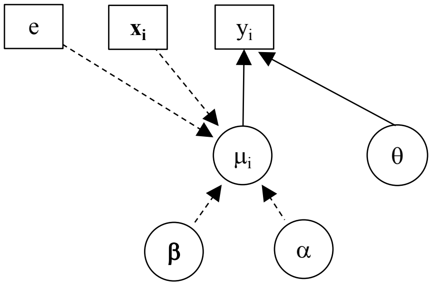

Figure 2. DAG for the negative binomial regression model. Dashed lines represent deterministic relationships and solid line represent stochastic relationships. Rectangles represent data and circles model parameters. Vector of values are represented in bold.

3. Poisson regression with zero inflated extension

Equation 3

$$y_i^0 \sim Bernoulli\ (\pi_i)$$

$$logit(\pi_i) = \alpha^0 + \sum_{1=k}^{K} \beta_K^0\, x_{i,k}^0$$

Equation 4

$$y_i \sim Poisson\ (\mu_i(1-\pi_i))$$

$$log(\mu_i) = \alpha + \sum_{1=k}^{K} \beta_K\, x_{i,K} + log(e)$$

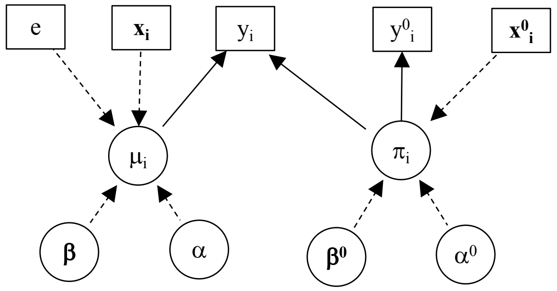

Figure 3. DAG for the Poisson regression model with zero inflation logistic extension. Dashed lines represent deterministic relationships and solid line represent stochastic relationships. Rectangles represent data and circles model parameters. Vector of values are represented in bold.

4. Negative binomial regression with zero inflated extension

Equation 5

$$y_i^0 \sim Bernoulli\ (\pi_i)$$

$$logit(\pi_i) = \alpha^0 + \sum_{1=k}^{K} \beta_K^0\, x_{i,k}^0$$

Equation 6

$$y_i \sim Negative\ Binomial\ (\mu_i(1-\pi_i), \theta)$$

$$log(\mu_i) = \alpha + \sum_{1=k}^{K} \beta_K\, x_{i,K} + log(e)$$

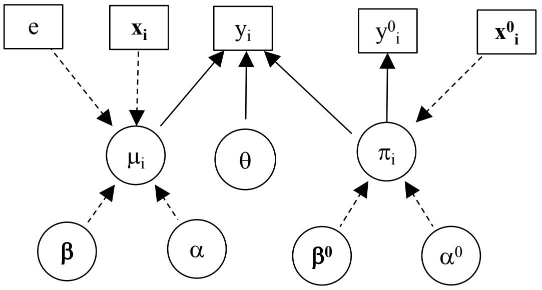

Figure 4. DAG for the negative binomial regression model with zero inflation logistic extension. Dashed lines represent deterministic relationships and solid line represent stochastic relationships. Rectangles represent data and circles model parameters. Vector of values are represented in bold.
